# Estimating the Cost and Carbon Output of Musculoskeletal Primary Care Management Decisions: A Retrospective Analysis of Electronic Health Records

**DOI:** 10.1002/hpm.3919

**Published:** 2025-03-22

**Authors:** Alex Braybrooke, Melissa Pegg, Rebecca Naylor, James Bailey, James Scott, Roanna Burgess, Dahai Yu, Simon Wathall, Kelvin P. Jordan, Robert Malcolm, Hayden Holmes, George Peat, Anirban Banerjee, Jonathan C. Hill

**Affiliations:** ^1^ School of Allied Health Professions and Pharmacy Keele University Keele UK; ^2^ Centre for Musculoskeletal Health Research Keele University Keele UK; ^3^ York Health Economics Consortium University of York York UK; ^4^ School of Medicine Keele University Keele UK; ^5^ Sandwell and West Birmingham NHS Trust West Bromwich UK; ^6^ Centre for Applied Health & Social Care Research (CARe) Sheffield Hallam University Sheffield UK

**Keywords:** carbon footprint, general practice, health economics, musculoskeletal disorders, primary health care

## Abstract

**Background:**

Healthcare accounts for up to 5% of worldwide carbon emissions and costs global economies an estimated $9 trillion annually. Primary care accounts for up to one‐fifth of all NHS carbon emissions, with musculoskeletal (MSK) pain accounting for 14%–30% of all primary care consultations.

**Method:**

A cost‐carbon calculator model was used to undertake a retrospective economic and environmental analysis of resource use for non‐inflammatory MSK pain primary care consulters. Data used to populate the model was derived from Electronic Health Records and patient surveys collected during The Multi‐level Integrated Data for Musculoskeletal Health Intelligence and ActionS GP Study. The model was utilised to estimate the mean (with 95%CI's) cost and carbon output per MSK consulter, while also examining variations at two levels: (a) the Primary Care Network (PCN), and (b) the consulter's index MSK pain site.

**Results:**

One thousand eight hundred seventy‐five individuals from 30 NHS primary care practices across 13 PCNs were eligible for EHR and survey data analysis. The mean carbon and cost output per person (over 6 months) was 46.91 kg CO_2_e (95% CIs; 45.02, 48.81 kg CO_2_e) and £182.65 (95% CIs; £178.69, £190.62), respectively, with substantial variation observed across PCNs. The resource category with the highest carbon footprint was consistently pharmacological intervention across all PCNs. Individuals who consulted for multisite/widespread pain and back pain had the highest mean carbon and cost output respectively.

**Conclusion:**

This is the first study, we are aware of, that presents data on both the environmental and economic impact of the primary care of non‐inflammatory MSK pain. Future work should focus on benchmarking the cost and carbon output of MSK care pathways and standardising methods that are implemented to influence sustainable practice and policy development.

**Summary**

- The carbon footprint and associated costs of MSK in primary care are relatively unknown
- A cost‐carbon calculator was used to estimate the economic and environmental impact of MSK care
- We estimated the mean carbon and cost output of healthcare resource use for an individual who consults a primary care clinician for non‐inflammatory MSK pain to be 46.91 kg CO_2_e (95% CIs; 45.02, 48.81 kg CO_2_e), which is the equivalent of driving 120 km by car and £182.65 (95% CIs; £178.69, £190.62) over the 6 months after an individual's index consultation
- Substantial variation was observed between primary care networks, far exceeding the variation by different MSK pain sites

**Plain Language Summary**

Why did we do this study?

- Healthcare services significantly contribute to global carbon emissions and costs. General practice makes up a large proportion of healthcare services within the UK's NHS. Musculoskeletal (MSK) conditions, such as back and joint pain, account for up to 30% of all GP consultations. By 2050 the NHS has pledged to achieve Net Zero, meaning that it aims to minimise the total volume of greenhouse gas emissions that it generates. To achieve this, all areas of healthcare must strive to establish more sustainable practices. Therefore, the reason for doing this research was to establish methodology to assess for variation in the carbon and cost output of care associated with MSK conditions. Likewise, we aimed to assess for variation in the carbon and cost output between primary care networks and between different MSK pain sites using real world data.

What did we do?

- In this study, we created a technique to calculate the environmental (carbon footprint) and economic (cost) impact of MSK care in the NHS general practice. We analysed data from health records and patient surveys involving 1875 individuals across 30 GP practices that were collected during the MIDAS GP project. The data extracted covered healthcare resources that are commonly associated with the management of MSK conditions. All resources came under the following categories: (1) GP or healthcare practitioner appointments, (2) Medication prescriptions, (3) Imaging/Scans, (4) Self‐management, (5) Urgent referrals, (6) Routine referrals, (7) Patient travel, and (8) Additional outcomes.

What did we find?

- On average, we estimated that caring for one MSK patient over 6 months resulted in 46.91 kg of CO_2_e and costs £182.65. However, there were noticeable differences between practices. For all the patients who presented with MSK pain, medication prescription was found to be the highest contributor to carbon emissions. Due to high levels of medication prescription, the care associated with patients who presented with multiple pain sites or widespread pain had the highest carbon output. Likewise, because of high imaging rates, care associated with back pain patients had the highest cost. It was not within this project's scope to map how differing levels of carbon and cost output affected patient outcomes.

What does it mean?

- This is the first study to combine both carbon and cost measurements for MSK care in primary care settings. The methods established within this study provide a framework for future research in this area. Additionally, future research should focus on creating benchmarks for sustainable and cost‐effective MSK care and implementing policies to reduce healthcare's environmental impact without compromising patient outcomes.

## 1 Introduction

Climate change is a global crisis, with the World Health Organisation (WHO) [1] predicting approximately 250,000 excess deaths by 2050 attributed to undernutrition, malaria, diarrhoea, and heat stress directly due to environmental changes. Sustainable development, defined as ‘development that meets the needs of the present without compromising the ability of future generations to meet their own needs’ [2], necessitates urgent and substantial contributions from society towards achieving Sustainable Development Goals (SDGs), that the United Nations has set out [3]. SDG three calls on healthcare systems to promote long‐lived good health and well‐being to people of all ages. However, by 2030, it is thought that the worldwide additional cost of ill human health directly related to environmental changes will be 1.1 trillion US dollars on global healthcare systems, roughly a 10% increase from current expenditure [4, 5]. Meanwhile, in the United Kingdom (UK) alone, it is estimated that by 2070 summers will be 6°C hotter and 60% drier [6]. In low and middle‐income countries (LMICs), the potentially devastating effects of climate change on vulnerable, densely populated areas, combined with a significant increase in the ageing population, are expected to substantially increase the burden that environmental changes will have on healthcare systems [7, 8]. Promoting sustainable human behaviour that can directly impact climate change and reduce its associated cost to healthcare systems is therefore high on the global political agenda [9].

Carbon dioxide (CO_2_) represents around 86% of all GHG emissions globally which is the reason why, at present, it is the universally accepted unit of measurement for measuring resource environmental impact [9]. The sum of direct and indirect GHG emissions associated with a product is referred to as the products' carbon footprint/output and is reported in megatons (Mt), tonnes (t), or kilogrammes (kg) of carbon dioxide equivalent (CO_2_^e^) (one megaton = one million tonnes or one billion kilogrammes) [10]. CO_2_^e^ compares the emissions from various greenhouse gases based on their global‐warming potential (GWP), by converting amounts of other gases (namely: methane (CH_4_), nitrous oxide (N_2_O), perfluorocarbons (PFCs), hydrofluorocarbons (HFCs), sulphur hexafluoride (SF_6_) and nitrogen trifluouride (NF_3_)) to the equivalent amount of carbon dioxide with an equivalent GWP [10, 11]. The 2020 Lancet Countdown on Health and Climate Change Report [12] identified the healthcare sector as one of 41 key drivers that can help with the mitigation of GHG emissions. The report [12] estimated that the healthcare sector is responsible for approximately 4.6% (2000 MtCO_2_e per annum) of all global GHG emissions, with additional evidence [13, 14] suggesting that the United Kingdom's (UK) National Health Service (NHS) alone emits 25 MtCO_2_e per annum, roughly equivalent to the total yearly GHG emissions of Croatia [15]. Furthermore, it has been reported that there is a large variation in GHG emissions between global healthcare systems, even when they have similar Healthcare Access and Quality Indexes (HAQ) [12]. For example, France, Japan, and the USA all have similar HAQ Indexes but have estimated annual GHG emissions of 350, 1220, and 1720 kg CO_2_e per capita, respectively [12]. Meanwhile, countries where climate change is likely to have greater consequences, such as India and Indonesia, have healthcare systems that are emitting substantially less GHG's [12].

In 2020, the NHS pledged to become the first national healthcare system worldwide to achieve net zero, meaning that by 2050 the healthcare service aims to achieve net zero emissions between the amount of GHGs generated compared with the amount that it contributes to removing from the atmosphere [16]. The GHG Protocol [17] classifies carbon emissions into three scopes that the NHS must tackle to achieve net zero. Namely, Scope 1: direct emissions from owner or directly controlled sources (including GHG emitted from NHS facilities, such as the use of fuel for heating); Scope 2: Indirect emissions from the generation of purchased energy (mostly electricity); and Scope 3: all other indirect emissions that occur in producing and transporting goods and service (Including emissions from NHS supply chains, and staff travel). In addition, the NHS carbon footprint plus [16] recognises an additional scope (scope 4) that encompasses patient and visitor travel to and from NHS services as well as medicines used within their homes. Despite significant progress in recent years, which has seen the NHS reduce its scope 1 emissions by 57% and its scope 3 emissions by 22% [16] current estimates suggest that the NHS will not meet its net‐zero targets [18]. Thus, highlighting the need for strategies that support further GHG emissions mitigation within healthcare services.

Primary care represents one of the largest proportions of healthcare service activity in the UK, with one‐fifth of NHS employees working within this sector [19]. In 2022 there were approximately 329 million primary care consultations within the NHS, across a wide range of professions including general practice (GP), physiotherapy, and first contact practitioners (FCPs) [19]. Nicolet et al. (2022) [20] undertook a retrospective analysis of 10 primary care practices in Switzerland and used a lifecycle assessment (LCA) to estimate that an average primary care consultation is responsible for emitting 4.8 kg CO_2_e. Accounting for between 14% and 30% of primary care consultations [21, 22] musculoskeletal (MSK) conditions cause individuals pain in and around their joints, bones, and muscles. Individuals with MSK conditions are most frequently managed in primary and community care, with recent evidence suggesting high levels of variation in care, that can burden resource use [23]. Similarly, they represent one of the largest contributors to years lived with disability [24], with recent evidence showing a substantial increase in their prevalence, most concerningly in LMICs [25]. Consequently, optimising care pathways to ensure individuals receive evidence‐based treatment is key to minimising the impact of MSK conditions on healthcare systems and the environment. To the best of our knowledge, there have not been any prior studies that have attempted to quantify the carbon output of primary care of MSK conditions, with a recent scoping review [26] highlighting the need for work in this area.

Similarly, a recent review by Burgess et al. (2020) [27] highlighted that there is a large variation in the costing methodology used within primary and community MSK care pathways. From the 22 studies included in the review, the authors concluded that high utilisation of lower‐cost resources (such as GP and physiotherapy visits) was driving the highest proportion of healthcare costs in this setting, rather than high unit cost items (e.g., MRI scans), with the review recommending the need for further scrutinisation of the cost‐effectiveness of entire MSK care pathway. Furthermore, the authors also recommended a need for improved and standardised methods to support the accurate capture and reporting of key cost drivers within community and primary healthcare.

The Multi‐level Integrated Data for Musculoskeletal Health Intelligence and ActionS GP Study (MIDAS GP study) [https://www.keele.ac.uk/midas/] [28], was a multi‐site GP observational cohort study designed to provide MSK health intelligence to healthcare commissioners, managers, and clinicians that can help reduce outcome variability between MSK primary care services and, therefore, improve healthcare delivery. The MIDAS GP Study collected real‐world evidence of MSK primary care treatment over 6 months from GP practices in North Staffordshire, UK. In addition, researchers from Keele University and the York Health Economics Consortium (YHEC) are collaborating on the MSK Pathways clinical trial [ISRCTN protocol registration number: 38,924,614][29] to test the clinical, cost, and carbon impact of a digital clinical decision support system called Orthopathway in the Birmingham and Solihull region, UK. As part of this trial, the team has developed an MSK pathways cost‐carbon calculator (hereafter ‘the model’) (Full model available in Supporting Information S1) that includes cost and carbon output values associated with the most frequently used resources in MSK healthcare.

Therefore, the objectives of this retrospective electronic health care record (EHR) analysis were:

1. To test the feasibility of populating an MSK pathways cost‐carbon model calculator using EHR and survey data collected during the MIDAS‐GP Study.
2. To estimate the average cost and associated carbon emissions of primary care management decisions for individuals who consulted a primary care clinician for non‐inflammatory MSK pain in the MIDAS GP study, at resource category (e.g. medication prescription) and individual resource (e.g. co‐codamol) level.
3. To describe the variation in the cost and carbon output of non‐inflammatory MSK pain management decisions (at resource category level) between a) healthcare providers (at Primary Care Network (PCN) level) and b) MSK pain site.

## 2 Methods and Data

### 2.1 Study Design

This study consisted of a retrospective health economic and environmental analysis of EHR and survey data from individuals who participated in the MIDAS GP study.

### 2.2 The MIDAS GP Study

The MIDAS GP Study recruited individuals who consulted a primary care clinician for non‐inflammatory MSK pain between September 2021 and June 2022, across 30 GP practices in 13 Primary Care Networks (PCNs) within Staffordshire, UK. Data from consenting participant's primary care EHR was collected to capture treatment and management decisions. Within the study, participants also completed a baseline and follow‐up survey (at three and 6 months after their index consultation), which collected data on patient outcomes, experiences, and self‐management options. The MIDAS GP study gained ethical approval from the Yorkshire and Humber–Leeds West Health Research Authority Ethics Committee in 2021 (REC Reference: RG‐0327–21), with full data collection methods of the study described elsewhere [28].

## 3 MSK Pathway Cost‐Carbon Model Development

The model was designed as part of a cluster randomised trial evaluating a digital healthcare technology, aiming to optimise the MSK primary care pathway [29]. The model was designed to enable experts to collaborate at scale and consolidate optimal clinical decision processes, standardise MSK treatment pathways, and thereby minimise inefficiencies in MSK care provision. As part of scoping the model structure, a targeted literature review was carried out to highlight the potential resource use, costs, and carbon impacts for the model pathways. The search strategy used in this process is detailed in Supporting Information S2. Inputs collected through this literature review were used to populate the model, while the over‐arching approach was informed by similar economic evaluations. The model structure maps out 62 key resources (full list Supporting Information S1), categorised into eight resource categories, mainly focussing on resources used in primary/community care and several resources used in secondary care across MSK care pathways. Table 1 describes the resources, resource categories, and cost and carbon output values used within the study (only resources that were feasible to populate), with references for the cost and carbon values used available in Supporting Information S1.

**TABLE 1:** Primary MSK care resource cost and carbon output estimates and mean resource use and carbon and cost output in the overall MIDAS GP cohort (*n* = 1875).

Resource category	Resource	Estimated carbon output (kg CO_2_e)	Estimated cost (£)	Resource use count per person (Mean, 95% CI's)	Mean carbon output per person (95% CI's) (kg CO_2_e)	Mean cost per person (95% CI's) (£)
Primary care appointments^a^	GP appointment–Face‐to‐face	6.00	41.00	0.93 (0.88, 0.97)	5.57 (5.30, 5.84)	39.00 (37.01, 40.91)
	GP appointment–Telephone	0.03	15.80	0.83 (0.79, 0.88)	0.03 (0.02, 0.03)	13.15 (12.42, 13.89)
	GP appointment–Video	0.02	41.13	0.01 (0.00, 0.01)	0.00 (0.00, 0.00)	0.26 (0.11, 0.41)
	FCP appointment–Face‐to‐face	6.00	19.26	0.21 (0.18, 0.23)	1.24 (1.09, 1.38)	3.97 (3.50, 4.43)
	FCP appointment–Telephone	0.03	7.18	0.10 (0.08, 0.11)	0.00 (0.00, 0.00)	0.74 (0.61, 0.86)
	FCP appointment–Video	0.02	18.86	0.00 (0.00, 0.00)	0.00 (0.00, 0.00)	0.03 (0.00, 0.06)
Diagnostic imaging^a^	Imaging–Xray	0.76	41.41	0.08 (0.06, 0.09)	0.06 (0.48, 0.07)	3.14 (2.63, 3.64)
	Imaging–Ultrasound	0.53	77.81	0.05 (0.04, 0.06)	0.03 (0.02, 0.03)	3.69 (2.87, 4.52)
	Imaging–CT scan	9.20	104.97	0.03 (0.02, 0.04)	0.30 (0.22, 0.39)	3.47 (2.54, 4.40)
	Imaging–MRI	17.50	188.11	0.12 (0.10, 0.14)	2.11 (1.77, 2.45)	22.67 (19.05, 26.30)
Additional outcomes^a^	Full blood count	0.12	9.03	0.08 (0.06, 0.0.9)	0.01 (0.01, 0.01)	0.68 (0.52, 0.85)
	Nerve conduction study	24.50	121.66	0.01 (0.01, 0.04)	0.00 (0.00, 0.00)	3.05 (1.48, 4.62)
	Steroid injection	2.53	31.84	0.01 (0.01, 0.02)	0.03 (0.02, 0.04)	0.39 (0.22, 0.56)
Medication prescription^a^	Paracetamol	4.43	1.34	0.36 (0.29, 0.43	1.59 (1.28, 1.90)	0.48 (0.39, 0.58)
	Codeine	4.43	1.06	0.22 (0.15, 0.28)	0.96 (0.68, 1.23)	0.23 (0.16, 0.29)
	Co‐codamol	4.43	2.94	0.92 (0.82, 1.02)	4.09 (3.64, 4.53)	2.71 (2.42, 3.01)
	Tramadol	4.43	2.90	0.19 (0.14, 0.23)	0.84 (0.64, 1.03)	0.55 (0.42, 0.67)
	Oxycodone	4.43	4.32	0.01 (0.00, 0.01)	0.03 (0.00, 0.06)	0.02 (0.00, 0.06)
	Buprenorphine	4.43	5.04	0.00 (0.00, 0.01)	0.01 (0.00, 0.03)	0.01 (0.00, 0.04)
	Buprenorphine transdermal patch	4.43	17.60	0.06 (0.03, 0.08)	0.25 (0.12, 0.37)	0.99 (0.48, 1.49)
	Morphine	4.43	5.31	0.11 (0.06, 0.16)	0.49 (0.16, 0.71)	0.59 (0.32, 0.86)
	Ibuprofen	4.43	3.12	0.13 (0.10, 0.17)	0.59 (0.45, 0.74)	0.42 (0.32, 0.52)
	Naproxen	4.43	4.29	0.45 (0.39, 0.51)	1.99 (1.75, 2.24)	1.93 (1.69, 2.17)
	Celecoxib	4.43	5.32	0.02 (0.01, 0.03)	0.08 (0.02, 0.14)	0.10 (0.03, 0.17)
	Etoricoxib	4.43	2.29	0.03 (0.01, 0.05)	0.14 (0.06, 0.22)	0.07 (0.03, 0.11)
	Amitriptyline	4.43	0.75	0.61 (0.53, 0.70)	2.72 (2.34, 3.11)	0.46 (0.40, 0.53)
	Gabapentin	4.43	3.35	0.46 (0.38, 0.54)	2.04 (1.69, 2.39)	1.54 (1.28, 1.81)
	Pregabalin	4.43	2.30	0.30 (0.24, 0.37)	1.35 (1.07, 1.62)	0.70 (0.56, 0.84)
	Nortriptyline	4.43	2.02	0.04 (0.02, 0.06)	0.16 (0.07, 0.25)	0.07 (0.03, 0.12)
	Duloxetine	4.43	2.72	0.20 (0.13, 0.26)	0.87 (0.57, 1.16)	0.53 (0.35, 0.71)
	Venlafaxine	4.43	4.03	0.06 (0.03, 0.09)	0.28 (0.14, 0.42)	0.25 (0.12, 0.38)
Urgent referrals^a^	Trauma and orthopaedics	22.00	158.62	0.04 (0.03, 0.05)	0.94 (0.73, 1.15)	6.77 (5.26, 8.28)
	Neurology	22.00	213.50	0.00 (0.00, 0.00)	0.05 (0.00, 0.09)	0.46 (0.01, 0.90)
	Rheumatology	22.00	165.18	0.01 (0.00, 0.01)	0.14 (0.06, 0.22)	1.06 (0.46, 1.56)
	Oncology	22.00	205.78	0.06 (0.05, 0.08)	1.41 (1.15, 1.67)	13.17 (10.73, 15.61)
	MSK triage service	22.00	6.25	0.01 (0.00, 0.01)	0.19 (0.09, 0.29)	0.01 (0.00, 0.01)
Routine referrals^a^	Orthopaedic	22.00	158.62	0.24 (0.22, 0.26)	5.33 (4.84, 5.81)	38.41 (34.90, 41.92)
	Rheumatology	22.00	165.18	0.02 (0.01, 0.02)	0.40 (0.27, 0.53)	3.00 (2.00, 4.00)
	Oncology	22.00	205.78	0.00 (0.00, 0.00)	0.00 (0.00, 0.00)	0.00 (0.00, 0.00)
	Neurology	22.00	213.50	0.01 (0.01, 0.02)	0.25 (0.14, 0.35)	2.39 (1.37, 3.41)
	Podiatry	22.00	93.37	0.00 (0.00, 0.01)	0.09 (0.03, 0.16)	0.40 (0.12, 0.67)
	Physiotherapy	22.00	100.47	0.11 (0.09, 0.13)	2.43 (2.08, 2.78)	11.10 (9.50, 12.70)
Self‐management^b^ options^a^	Brace support or splint	0.00	0.00	0.16 (0.15, 0.18)	0.89 (0.80, 0.98)	—
	Online info/Advice	0.03	0.00	0.17 (0.15, 0.19)	0.01 (0.00 0.01)	—
	Equipment, aids, or adaptions	5.50	0.00	0.15 (0.13, 0.16)	0.80 (0.72, 0.89)	—
	Outdoor activity/Home exercise	0.00	0.00	0.51 (0.48, 0.55)	0.00 (0.00, 0.00)	—
	Information leaflet	0.07	0.00	0.11 (0.09, 0.12)	0.01 (0.01, 0.01)	—
	Vitamins or supplements	4.43	0.00	0.27 (0.24, 0.30)	1.20 (10.8, 1.31)	—
	Peer support group	1.60	0.00	0.02 (0.01, 0.02)	0.03 (0.02, 0.04)	—
Travel	Travel to a primary care appointment^c^	1.32	0.00	—	1.50 (1.39, 1.52)	—
	Travel to a secondary care outpatient appointment^c^	6.8	0.00	—	3.47 (3.25, 3.67)	—

*Note:* References for cost and carbon values are available within in original model in Supporting Information S1.

^a^ Data extracted from individual's EHR.

^b^ Data extracted from MIDAS participants baseline satisfaction survey.

^c^ Calculated on the basis that 1 km of travel by car emits 0.4kgCO_2_e, and the average distance travelled to primary care appointments and secondary care outpatient appointments is 3.3 and 17.0 km, respectively.

### 3.1 Acquisition of Carbon Data Values and Assumptions Made

Currently, there is a lack of appropriate cost and carbon data available across MSK pathway common resource use [26, 27]. Carbon emissions data for resource use have been acquired from published data that relies on appropriate scope and system boundary methods to quantify carbon emissions of resources. The carbon data was sourced from publications that either applied a top‐down approach (environmentally extended input‐output method based upon the monetary cost of items) [30] or a process‐based method [31] using activity data such as energy or materials use. International standards have been used including the greenhouse gas emissions protocols to ensure carbon data quality and to reduce the risk of truncation error; whereby processes may be omitted, or the so‐called ‘hidden’ sectors may be overlooked [2]. Consequently, several assumptions are clearly defined within the model. Firstly, it was assumed that FCPs and self‐referral physiotherapy appointments in primary care have the same carbon output as GP appointments. Secondly, it was assumed that all medication prescriptions have the same carbon footprint as each other, with the following top‐down approach used: 1) The NHS carbon footprint in 2020 was 24.9 million tonnes of CO_2_e; 2) Medication make up approximately 20% of the total NHS carbon footprint = 4.98 million tonnes CO_2_e; 3) There were 1,123, 515, 663 community prescriptions in England in 2020; 4) Therefore 4,980, 000, 000/1,123, 514, 663 = 4.4325 kg CO_2_e per prescription. Thirdly, all outpatient appointments were assumed to have the same carbon output. Likewise, as a result of not having individual patient travel data, national averages were used to estimate carbon emissions for both individual's travel to primary care and secondary care outpatient appointments. Finally, for secondary care outpatient referrals we did not assume how many appointments individuals would attend and, therefore, for services such as physiotherapy and podiatry, we based our analysis on the principle that ‘one primary care referral = one secondary care appointment’.

### 3.2 Electronic Health Care Record and Patient Survey Data Extraction

Two authors matched Systematised Nomenclature Medicine Clinical Terms (SNOMED CT) codes (Codes used in the UK primary care to record morbidities and processes of care) from EHR data against resources within the model (Full list of codes available on request). The only explicit criteria for resource population were whether SNOMED CT codes were available within the database and if the resource use was asked about in the survey. After this, they developed a data query to extract count values for resources included in the model. The data extraction query captured MSK consulter's EHR data for 6 months (180 days) from their index consultation with a primary care practitioner following a primary care consultation for a non‐inflammatory MSK condition, using a pre‐defined diagnostic and symptom code list [32]. Finally, survey data collected from participants in the MIDAS GP study 3 and 6 months after their index primary care consultation was used to calculate the self‐management resources that they used for their MSK condition, over the same 6‐month period. The details of the methods used to calculate count values for self‐management resources from the two patient surveys can be found in Supporting Information S3.

### 3.3 Data Management and Analysis

In the model, resources were split into the following eight categories that represent different aspects of MSK condition management: primary care appointments; diagnostic imaging; additional outcomes; medication prescription; urgent referrals; routine referrals; self‐management; and patient travel. Table 1 presents the resources within each category and their associated carbon and cost output. The list of primary care resources within the model was determined a priori by a group of primary care experts involved in the MSK Pathways trial. The health economic analysis was taken from the perspective of the economic cost (£) of each resource to NHS England. Meanwhile, the environmental analysis (kg CO_2_e) examined an estimation of the carbon emissions impact of the resources used. The costs of patient travel and self‐management were not considered to be directly attributable to the NHS. However, since carbon output attributed to patient travel and self‐management contributes to carbon emissions, they were, included in the environmental assessment.

For the analysis, the model was used to calculate the mean cost and carbon output per patient within the overall cohort and at PCN level. The model also enabled us to calculate the mean cost and carbon output of individual resources and by resource category. Having completed this stage, we mapped mean cost and carbon values against participant characteristics data within Microsoft Excel and then exported the data to SPSS for the next part of our analysis. Here, the variation in cost and carbon output of clinical resource use was mapped across the local area by calculating means and 95% confidence intervals for each resource category within each PCN. Thereafter, the highest cost and carbon‐emitting resources within each resource category were calculated in the overall cohort. Finally, data from individuals were segregated by index consultation pain site (extracted from EHRs) to enable a comparison of the cost and carbon output of various MSK pain sites.

## 4 Results

A total of 1875 MSK consulters responded to the MIDAS GP survey and gave consent for data linkage to their primary care EHR. Tables 2 and 3 show the participant characteristics of the 1875 participants in the MIDAS study and selected population demographic variables for each of the 13 PCNs.

**TABLE 2:** MIDAS participant and PCN characteristics.

PCN	Number of participants, *n* (% of overall)	Age, mean (SD)	Females, *n* (%)^b^	MSK‐HQ score at intake (mean, SD)	PCN total population (raw list Jan 22)^a^
Overall	1875	57.7 (15.5)	1222 (65.7)	25.3 (10.7)	475,012
PCN 1	96 (5.1)	54.3 (14.5)	60 (63.2)	22.2 (11.0)	40,621
PCN 2	131 (7.0)	52.8 (15.8)	86 (66.2)	22.4 (10.3)	33,145
PCN 3	122 (6.5)	50.6 (14.7)	83 (68.6)	23.2 (10.3)	42,022
PCN 4	177 (9.4)	59.8 (14.7)	117 (66.1)	26.1 (11.3)	50,087
PCN 5	189 (10.1)	57.7 (16.0)	129 (68.3)	24.8 (10.0)	36,985
PCN 6	49 (2.6)	65.9 (15.3)	31 (63.3)	29.0 (11.6)	37,294
PCN 7	145 (7.7)	57.2 (16.0)	96 (66.7)	27.3 (11.2)	40,836
PCN 8	164 (8.7)	61.4 (14.7)	98 (60.5)	25.6 (11.0)	35,260
PCN 9	214 (11.4)	62.6 (14.6)	142 dye (67.9)	26.9 (10.8)	42,501
PCN 10	149 (7.9)	54.4 (15.8)	84 (56.4)	24.3 (9.2)	37,108
PCN 11	244 (13.0)	57.3 (14.3)	161 (66.3)	25.1 (10.4)	48,702
PCN 12	131 (7.0)	58.5 (14.7)	90 (69.2)	27.0 (11.4)	32,585
PCN 13	64 (3.4)	60.5 (16.1)	45 (71.4)	25.0 (9.9)	38,702

^a^ Data extracted from IMD 2019 report.

^b^ Difference in reporting of gender frequencies between this and other MIDAS GP papers is due to 14 missing gender cells within MIDAS EHR dataset.

**TABLE 3:** Pain site at index consultation in the overall cohort (*n* = 1875).

Pain site at index consultation	*n* (%)
Back	546 (29.2)
Hip/Knee	496 (26.5)
Shoulder/Elbow	267 (14.2)
Foot/Ankle	145 (7.7)
Multisite/Widespread	117 (6.2)
Neck	107 (5.7)
Unspecified	99 (5.3)
Hand/Wrist	97 (5.2)

### 4.1 Result 1: Feasibility of Populating the Cost/Carbon Model

Searches within primary EHR‐sourced data enabled 51 (82.23%) of the 62 resources to be populated within the original version of the model (Table 1). The 11 resources that were not possible to populate were attendance to A&E, inpatient hospital stays, surgical intervention (both elective and non‐elective), travel to secondary care by ambulance, sports and exercise medicine referral (both routine and urgent), as well as the self‐management resources: acupuncture, exercise (gym), and Fitbit/wearable device. It was not possible to populate the first five resources because this would have required additional access to secondary care records. For routine and urgent sports and exercise medicine referrals, no SNOMED CT codes were identified within the Keele University database, and the MIDAS GP study survey data did not provide information about how frequently individuals used gyms or wearables for their MSK condition and did ask individuals about acupuncture.

### 4.2 Result 2: Carbon Output

**FIGURE 1:**
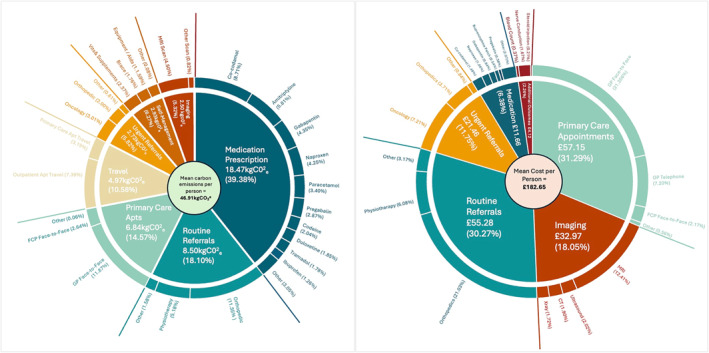
The mean carbon and cost output (% of total) per person within the overall cohort by resource category.

**FIGURE 2:**
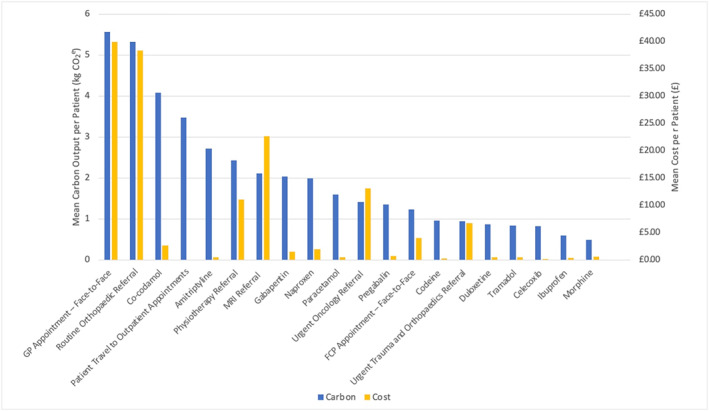
The top 20 highest emitting carbon resources and their associated cost in the overall cohort.

**FIGURE 3:**
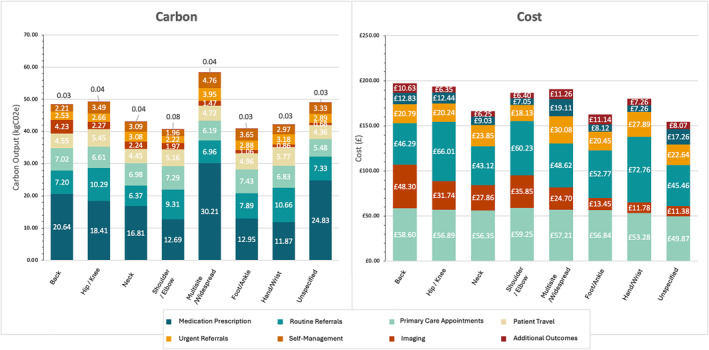
The mean carbon and cost output per person within the overall cohort by pain site at index consultation.

Overall, we estimated the mean carbon output of an MSK management, per person, in primary care over 6 months to be 46.91 kg CO_2_e (95% CIs; 45.02, 48.81) (Figure 1). Table 4 shows the mean (with 95% CI's) resource category carbon emissions in the overall cohort at PCN level, whilst Figure 1 visualises this. Among the overall cohort and within each PCN, medication prescription was consistently the highest resource category with the highest carbon output, producing an average of between 12.83 and 25.10 kg CO_2_e per person across PCNs, which accounted for 27.98%–45.05% of all GHG emissions. In the overall cohort, this was followed by routine referrals (8.50 kg CO_2_^e^, 18.10%) and primary care appointments (6.84 kg CO_2_e, 14.57%). Meanwhile, the highest mean emitting individual resources (Figure 2) in the overall cohort were face‐to‐face GP appointments (5.57 kg CO_2_e, 11.87%), routine orthopaedic referral (5.33 kg CO_2_e, 11.36%), co‐codamol prescription (4.09 kg CO_2_e, 8.71%), travel to outpatient appointments (3.47 kg CO_2_e, 7.39%), and amitriptyline prescription (2.73 kg CO_2_e, 5.81%). Individuals who consulted for multisite/widespread pain had the highest mean carbon output (58.83 kg CO_2_e), followed by hip/knee pain consulters (49.22 kg CO_2_e), and back pain consulters (48.40 kg CO_2_e) (Figure 3). Medication prescriptions accounted for a greater proportion of carbon emissions within multisite/widespread consulters (51.82%) than other specified MSK pain sites (28.16%–42.65%). However, medication prescription did account for 50.75% of GHG emissions in individuals who had an unspecified pain site at index consultation.

**TABLE 4:** Mean carbon output per person in the overall cohort and at PCN level by resource category.

PCN	Resource category (mean carbon output (95% CI; lower bound, upper bound)) (kg CO_2_e)								
	Primary care appointment	Diagnostic imaging	Additional outcomes	Medication prescription	Urgent referrals	Routine referrals	Self‐management	Travel	Total
Overall	6.84 (6.53, 7.13)	2.50 (2.15, 2.84)	0.04 (0.03, 0.05)	18.47 (17.18, 19.77)	2.73 (2.36, 3.08)	8.50 (7.87, 9.12)	2.93 (2.73, 3.13)	4.97 (4.72, 5.21)	46.91 (45.02, 48.81)
1	10.89 (9.20, 12.59)	4.24 (2.46, 6.01)	0.03 (0.00, 0.08)	25.10 (19.07, 31.13)	4.58 (2.45, 6.72)	7.79 (4.76, 10.82)	2.81 (2.08, 3.53)	6.22 (4.92, 7.51)	61.63 (52.01, 71.18)
2	6.83 (5.36, 8.30)	3.33 (1.68. 4.99)	0.05 (0.00, 0.11)	23.98 (18.88, 29.07)	4.03 (2.34, 5.72)	8.06 (5.60, 10.52)	3.42 (2.61, 4.23)	5.23 (4.20, 6.26)	54.58 (47.20, 62.56)
3	8.47 (7.00, 9.94)	1.73 (0.78, 2.67)	0.04 (0.00, 0.10)	18.30 (11.05, 25.55)	2.70 (1.22, 4.19)	7.75 (5.49, 10.02)	2.45 (1.76, 3.14)	5.09 (4.23, 5.96)	46.50 (37.67, 55.34)
4	7.15 (6.30, 8.00)	3.02 (1.70, 4.35)	0.05 (0.00, 0.09)	17.27 (13.09, 21.45)	2.86 (1.70, 4.01)	10.69 (8.49, 12.89)	3.56 (2.84, 4.29)	5.75 (4.92, 6.59)	50.31 (44.49, 56.13)
5	6.52 (5.81, 7.24)	2.53 (1.44, 3.61)	0.05 (0.01, 0.10)	15.94 (12.32, 19.56)	1.63 (0.80, 2.46)	5.59 (3.96, 7.21)	2.93 (2.29, 3.57)	3.66 (3.02, 4.31)	38.80 (33.32, 44.28)
6	7.36 (5.82, 8.91)	1.68 (0.00, 3.43)	0.03 (0.01, 0.06)	12.83 (6.69, 18.99)	3.59 (1.23, 5.95)	11.22 (6.73, 15.72)	2.97 (1.85, 4.09)	6.20 (4.68, 7.71)	45.86 (36.72, 55.00)
7	6.90 (5.71, 8.10)	3.28 (1.90, 4.65)	0.00 (0.00, 0.00)	16.16 (11.50, 20.81)	3.03 (1.71, 4.35)	8.19 (6.20, 10.19)	2.62 (2.00, 3.24)	4.98 (4.12, 5.84)	44.17 (37.85, 51.49)
8	8.25 (7.26, 9.24)	2.38 (1.32, 3.44)	0.02 (0.00, 0.05)	17.02 (13.32, 20.72)	0.94 (0.25, 1.63)	10.73 (8.25, 13.20)	2.69 (1.98, 3.39)	5.42 (4.53, 6.30)	47.43 (41.31, 53.54)
9	6.15 (5.40, 6.89)	1.66 (0.60, 2.72)	0.02 (0.00, 0.04)	16.40 (13.00, 19.80)	2.26 (1.27, 3.25)	10.69 (8.69, 12.70)	3.07 (2.47, 3.68)	5.35 (4.63, 6.07)	45.58 (40.19, 51.00)
10	8.98 (7.95, 10.02)	2.41 (1.27, 3.54)	0.09 (0.01, 0.12)	22.21 (16.78, 27.64)	3.10 (1.86, 4.34)	8.86 (6.52, 11.20)	2.44 (1.77, 3.10)	5.67 (4.79, 6.55)	53.67 (46.22, 61.12)
11	4.12 (3.41, 4.84)	2.35 (1.35, 3.34)	0.04 (0.02, 0.07)	20.12 (16.31, 23.92)	2.70 (1.60, 3.80)	8.11 (6.44, 9.79)	3.01 (2.42, 3.60)	3.58 (3.58, 4.90)	44.66 (39.23, 50.08)
12	5.30 (4.21, 6.39)	2.25 (1.15, 3.35)	0.06 (0.00, 0.13)	17.38 (13.29, 21.47)	2.02 (0.82, 3.21)	5.54 (3.82, 7.26)	2.89 (2.12, 3.66)	3.49 (2.73, 4.26)	38.87 (32.84, 44.91)
13	4.25 (3.34, 5.16)	1.33 (0.04, 2.62)	0.04 (0.00, 0.12)	15.85 (10.07, 21.64)	5.50 (2.91, 8.10)	6.53 (3.65, 9.42)	3.03 (1.84, 4.22)	4.65 (3.44, 5.85)	41.13 (33.15, 49.11)

Interestingly, there were different driving forces for why individual resources had high mean carbon emissions. For example, GP appointment (face‐to‐face), which was the highest mean carbon emitting resource (5.57 kg CO_2_e), was also the highest mean utilised resource (mean resource use = 0.93 [95CI's; 0.88, 0.97]), however, had a relatively low associated unit carbon output (4.43 kg CO_2_e). In contrast, routine orthopaedic referral, which was the second highest mean carbon emitting resource, had much higher associated carbon emissions (22.00 kg CO_2_e) but was the 10th most frequently used resource (mean resource use = 0.24 [95 CI's; 0.22, 0.26]).

### 4.3 Result 3: Costs

We estimated the mean cost of MSK primary care per person to be £182.65 (95% CIs; 178.69, 190.62) (Table 1), with Table 5 showing the mean cost per person with 95% confidence intervals for each resource category within the overall cohort and at PCN level. Primary care appointments accounted for the resource category with the highest associated cost within the overall cohort (£57.15; 95% CIs = 55.08, 59.23) and in all but one PCN (PCN 13), with the estimated cost ranging from £40.43‐£73.84. The five resources with the highest mean associated cost per person in the overall cohort were: face‐to‐face GP appointments (£39.00, 21.35%), routine orthopaedic referrals (£38.41, 21.03%), MRI referrals (£22.67, 12.41%), urgent oncology referral (£13.17, 7.21%), and GP telephone appointments (£13.15, 7.20%) (Figure 2). Back pain consulters had the highest associated cost among different pain sites (£193.55), followed by hip/knee (£189.56), and multisite/widespread consulters (£186.73) (Figure 3).

**TABLE 5:** Mean cost output per person in the overall cohort and at PCN level by resource category.

PCN	Resource category (mean resource cost output (£) (95% CI; lower bound, upper bound))						
	Primary care appointment	Diagnostic imaging	Additional outcomes	Medication prescription	Urgent referrals	Routine referrals	Total
Overall	57.15 (55.08, 59.23)	32.97 (29.11 36.84)	4.12 (2.44, 5.89)	11.66 (10.68, 12.64)	21.46 (18.52, 24.40)	55.28 (51.17, 59.39)	182.65 (178.69, 190.62)
1	73.84 (62.91, 84.78)	52.26 (33.31, 71.72)	0.71 (0.00, 1.46)	14.37 (10.93, 17.80)	35.72 (19.02, 52.42)	53.28 (31.98, 74.58)	230.18 (190.57, 269.79)
2	58.58 (50.22, 66.95)	38.68 (20.29, 57.07)	2.45 (0.32, 4.58)	15.12 (10.70, 19.54)	32.54 (18.17, 46.90)	52.48 (36.11, 68.84)	199.85 (166.39, 233.31)
3	61.22 (51.28, 71.16)	23.54 (12.04, 35.04)	0.67 (0.00, 1.42)	13.74 (7.74, 19.73)	24.91 (11.17, 38.66)	53.69 (37.90, 69.47)	177.77 (149.88, 205.66)
4	59.71 (52.89, 66.53)	38.83 (24.45, 53.20)	0.79 (1.33, 1.46)	12.13 (7.92, 16.34)	22.46 (13.17, 31.75)	68.12 (54.08, 82.16)	202.04 (174.92, 229.16)
5	48.21 (43.29, 53.14)	35.00 (23.09, 46.92)	9.64 (1.23, 18.04)	9.49 (7.26, 11.71)	12.19 (5.42, 18.69)	37.31 (26.31, 48.32)	151.84 (127.21, 176.48)
6	59.67 (48.57, 70.77)	20.88 (1.93, 39.82)	24.37 (4.60, 44.14)	7.61 (3.29, 11.93)	31.81 (10.79, 52.82)	73.65 (43.54,103.77)	217.99 (174.37, 261.61)
7	60.95 (52.32, 69.59)	43.29 (27.85, 58.73)	0.84 (0.00, 2.50)	9.16 (6.74, 11.59)	23.81 (13.04, 34.58)	53.50 (40.03, 66.98)	191.56 (159.87, 223.25)
8	66.83 (59.20 74.46)	33.93 (21.70, 46.15)	1.21 (0.00, 2.84)	10.52 (7.93, 12.13)	7.63 (1.99, 13.27)	67.36 (51.42, 83.30)	187.48 (160.65, 214.31)
9	55.12 (49.75, 60.48)	24.97 (12.93, 37.00)	0.40 (0.05, 0.76)	10.23 (7.76, 12.71)	20.53 (11.47, 29.59)	63.53 (51.10, 75.96)	174.78 (151.35, 198.21)
10	63.83 (56.78, 70.88)	30.20 (17.79, 42.60)	17.13 (0.68, 33.57)	13.11 (9.14, 17.07)	23.96 (13.99, 33.93)	54.55 (39.40, 69.16)	202.78 (173.82, 231.74)
11	46.80 (41.39, 52.21)	28.69 (17.88, 39.50)	2.07 (1.36, 2.79)	12.52 (9.99, 15.05)	19.11 (10.78, 27.43)	55.71 (44.19, 67.24)	164.90 (142.65, 187.65)
12	55.08 (46.78, 63.37)	33.84 (20.91, 46.76)	4.65 (0.95, 8.35)	12.71 (8.83, 16.59)	15.61 (6.18, 25.04)	40.48 (27.93, 53.02)	162.36 (136.16, 188.56)
13	40.43 (34.46, 46.39)	19.40 (4.88, 33.93)	0.50 (0.00, 1.49)	9.33 (5.62, 13.04)	35.83 (16.92, 54.73)	46.49 (25.76, 67.22)	151.98 (118.22, 185.73)

Similar to carbon emissions, there were different drivers behind high‐costing resources. In the top five highest‐costing individual resources, face‐to‐face GP appointments and GP telephone appointments were all driven by the high resource use within the population. Meanwhile, orthopaedic, MRI and urgent oncology referrals were driven by the high individual unit cost.

### 4.4 Result 4: Variation in Carbon and Cost Output

Substantial variation was observed in terms of the cost and carbon output across PCNs. The greatest variation between the lowest and highest mean carbon footprint (within resource categories) per person across the PCNs was seen in additional outcomes (9‐fold variation), urgent referrals (5‐fold variation), and imaging (3‐fold variation). However, due to the low resource use (breakdown available in Table 1) and associated carbon footprint (0.00–0.09 kg CO_2_e) of additional outcomes, there was, therefore, a very small difference between the absolute carbon footprint of additional outcomes across PCNs, meaning that any variation made little impact on PCNs overall carbon footprint. In contrast, variation in the carbon output of urgent referrals made a greater contribution to a PCNs overall carbon footprint. Additionally, amongst PCN's overall carbon output, there was a 1.5‐fold variation seen between the PCN with the lowest carbon output and the PCN with the highest.

For costs, the greatest variation between the highest and lowest associated cost (within resource categories) across PCNs was seen in additional outcomes (25‐fold variation), urgent referrals (5‐fold variation), and primary care appointments (2‐fold variation) (Table 4). High‐cost resources, such as nerve condition studies (Estimated cost = £121.66) highly skewed the variation seen within the additional outcomes resource category, which was not the case within urgent referrals, and primary care appointments, as individual resources had much similar estimated associated costs. As with carbon output, there was a 1.5‐fold variation seen between the lowest and highest overall cost between the PCNs.

Variation was also observed according to the pain site at index consultation. For carbon emissions, there was approximately a 2‐fold variation in the carbon output of diagnostic imaging referral between back pain consulters (4.23 kg CO_2_e) than any other body site (0.68 kg CO_2_e–2.27 kg CO_2_e). Meanwhile, for costs, the high cost associated with individuals who consulted for back pain could be partly attributed to high costs associated with diagnostic imaging (£48.30, 24.97%), which was 1.4 times greater than any other MSK pain site location at index consultation (shoulder/elbow = £35.85, 19.61%; hip/knee = £31.74, 16.74% of total).

## 5 Discussion

A key finding of this study is that medication prescription appears to account for the largest proportion of carbon output in the primary care management of non‐inflammatory MSK conditions but carries a relatively low cost. In addition, the top‐down environmentally extended input‐output (EEIO) method used to calculate total carbon emissions of medicines was used to determine the carbon cost of individual medicine prescriptions (a generic functional unit). It is recognised this approach lacks specificity and detail [33]. Therefore, it is likely, that the quantified carbon cost of low‐cost pharmaceuticals (e.g., Opioid analgesia), using the EEIO method, is underestimated. Moreover, the high prevalence of medication amongst MSK consulters within this population is concerning, given that the long‐term prescription of analgesia is currently not indicated in many MSK conditions [34, 35, 36], has limited supporting evidence for improving patient outcomes [37], and can cause long‐lasting harm to several organ systems [38]. Additionally, 80% of pharmaceutical pollution is concerned with the use of generic medicines, suggesting the disproportionate contribution of pharmaceutical environmental impact within healthcare [32]. In light of this, the true impact of medication prescription on the environment is likely to be greater than other resources [37], given that up to 90% of the medicines we consume end up in our wastewater, meaning that these pollutants affect the health of wildlife, contributing to biodiversity loss [39]. Furthermore, medications also drive antimicrobial resistance, one of the greatest global threats to human health [40, 41]. This study represents valuable evidence in assisting in the future development of policy and practice to help ensure the evidence‐based assessment and management of MSK conditions in primary care, whilst assisting in the mitigation of unnecessary pharmacology‐associated pollution within the healthcare sector. However, it must be acknowledged that this work did not assess the impact of resource use and carbon/cost output on service users' outcomes and experiences, and therefore, future work is needed to optimise resource use and sustainable practice, whilst ensuring individuals receive positive healthcare outcomes and experiences [40, 42].

Secondly, this study's feasibility findings can be used as a valuable tool by researchers, commissioners, and clinicians in undertaking carbon footprint analysis of MSK healthcare pathways, as we have shown it is possible to perform such analyses from EHRs and survey data to populate a cost‐carbon model. We have also highlighted that further work is required to link secondary care EHR that would allow for a more extensive MSK pathway analysis, as well as the need for accurate service user travel data to be collected.

Thirdly, we have estimated the average carbon emissions of MSK primary care clinical decisions (over 6 months) to be 46.69 kg CO_2_e, which is equivalent to driving approximately 120 km in a small car [42], with the three highest driving forces behind this being GP face‐to‐face appointments, routine orthopaedic referrals, and co‐codamol prescription. Fourthly, we estimated the average associated cost of MSK condition management to be £182.65 per person, with the main contributor to this cost being face‐to‐face consultations with a healthcare practitioner (GP or FCP). This cost is relatively low in comparison to some of the associated costs with secondary care interventions for MSK conditions (e.g., Elective, and non‐elective surgical intervention and inpatient stay = £9491.70 and £5292.41 respectively), and therefore, highlights the need for optimal care pathways which can minimise the need for secondary care interventions for MSK conditions.

Finally, there was substantial variation observed between PCNs for both cost and carbon output. The greatest variation in mean carbon and cost output per person between PCNs was seen in additional outcomes. Similarly, variation was also observed in terms of the cost and carbon output of individuals who consulted for different MSK pain sites. The variation in both carbon and cost output between local areas could have potentially significant implications for the UK's NHS's long‐term net zero plan if this variation is consistent across MSK care pathways, with evidence suggesting that such variation also exists within other areas of healthcare [23, 43]. Consequently, the authors propose that other care pathways would benefit from similar additional outcomes and methods used within this research.

To the best of our knowledge, this is the first study that has undertaken both an environmental and economic analysis of primary care assessment and management decisions for MSK decisions using EHR and survey data. This research addressed the need to undertake accurate environmental and economic analyses of MSK care pathways as highlighted by a recent scoping review that identified 24 studies that evaluated the carbon emissions of MSK healthcare, of which all included studies were in the context of either orthopaedic surgery or orthopaedic related hospital inpatient stays [26]. Additionally, and more concerning, there is a dearth of literature that assesses the economic and environmental impact of primary care in LMICs [44].

Despite this, there is a significant body of evidence from high‐income countries that have looked at the cost and carbon output of the healthcare sector as a whole or within specific healthcare disciplines from which meaningful comparisons can still be drawn. Nicolet et al., 2019 [20] looked at primary care consultations as a whole and estimated that the mean primary care consultation is 4.8 kg CO_2_e that was based on a lifestyle cycle assessment of 10 Swiss GP practices. Unlike our study, the Swiss study [20] did not assess the carbon emissions associated with clinical decision‐making, including medication prescription and diagnostic imaging referral, measuring the carbon output of a single primary consultation, rather than over a time horizon of 6 months hence the difference in carbon output estimates. Additionally, the study by Nicolet et al. [20], attributed 45.7% of all carbon emissions to service user and staff travel. The difference in study findings is noteworthy, since we found service user travel contributed 10.63% of emissions. This in part, could be explained by the difference in service user commute distance between British (3.3 km) and Swiss (5.5 km) primary care consulters.

Notwithstanding, air pollution associated with car travel, for example, is widely reported as contributing to human ill health. This difference highlights the need for future studies to collect survey data around the mode and distance that individuals travel to healthcare appointments to enable in‐depth environmental analyses. A potential solution to assist in the mitigation of carbon emissions associated with service user travel is the use of digital technologies, such as telehealth and clinician decision support tools, that can be used to help streamline healthcare pathways. However, it must also be acknowledged that digital health solutions are increasing energy use. For example, recent estimates have suggested energy use required to power artificial intelligence (AI) has increased by 48% within 5 years [45]. This has led to a recent urgent call by NHS England for robust evidence that assesses whether digital technologies truly increase or decrease a healthcare service's carbon footprint [46] and to assess their impact on healthcare quality [47].

This study aligns with several studies reporting variation among clinical management resource use for MSK conditions. Sajad et al. (2021) [23] reported a 30‐fold variation in MRI requests between GP practices for back pain consulters, suggesting that even when seeing individuals with similar symptom severity, clinicians are choosing different management options. Similarly, the use of clinical decision‐making influencing behavioural change techniques, including social support and restructuring physical environments, have been shown to reduce the carbon emissions associated with clinical activity across a range of healthcare settings [48], inferring that decision‐making prompts can be effective at optimising healthcare pathways, especially when the time lengths of consultations are limited. Implications of this variation may potentially greatly impact the NHS's capacity to meet its net‐zero target by 2050, with further work warranted to investigate the drivers behind this variation.

In terms of financial cost, Burgess et al. (2019) [27], reported that the resources with the highest mean cost per person in MSK care are GP consultations, followed by outpatient/medical specialist visits, and physiotherapy visits. Findings within the study are similar, with face‐to‐face GP appointments and routine orthopaedic referrals being the two largest contributors to mean cost output. In contrast, this study reported oncology referral to be the top five contributors to mean cost, something which was not found to be the case in the Burgess et al. (2019) [27] review. Within the overall cohort, our study found the mean urgent oncology rate to be 0.06 per person. This rate appears to be relatively consistent with existing evidence from NHS England, who report that in the UK the mean oncology referral rate per consultation from primary care is 4% [49]. The slightly higher referral rate within our population could potentially be explained by the known high levels of deprivation and high‐impact chronic pain within the North Staffordshire area [50]. Furthermore, due to the nature of EHR data extraction techniques used within this study, it may have been possible that an individual was referred to oncology after a follow‐up primary care appointment that was not for an MSK condition.

## 6 Conclusions

The main strength of this study is the novel approach that has been used to undertake both an environmental and economic analysis of the primary care of MSK conditions, that can be used to support the future development of sustainable policy and practice worldwide. Furthermore, within this approach we have addressed all three pillars of sustainability, namely: 1) social sustainability, by assessing the variation in resources that individuals across a local area receive for MSK conditions; 2) financial sustainability, by assessing the cost of resource use to the NHS; and 3) environmental sustainability, by assessing the carbon cost of resource use. Likewise, the cost and carbon values associated with resources were developed as part of a pragmatic literature search during the development of a robust cost‐carbon calculator model. Additionally, we extracted data from multiple sources (EHR and survey) allowing for self‐management resources to be incorporated into our analysis, something which would have not been possible when solely using EHR data.

However, this piece of work is not without limitations. Firstly, our calculations are based on a pre‐defined list of resources that authors regarded as being the most relevant to the MSK condition management. This list was compiled as part of a collaboration between Keele University and YHEC and was done so in consultation with a broad range of healthcare experts, including primary care clinicians, services managers, commissioners, primary care researchers, and health economists. Despite this comprehensive approach, there will be elements of data source uncertainty due to the heterogeneity of study methods, limiting the reliability and generalisability of findings. Findings may also be restricted by several methodological options, resulting in scenario uncertainty. For example, there may be a truncation error where the results reported underestimate the true GHG emissions generated, attributable to the chosen system boundary that determines the carbon emissions factors of functional units (resource use). Furthermore, the carbon emissions of medication were estimated using a top‐down approach based on medication use as a proportion of the total NHS carbon footprint [16]. This is unlikely to consider the disproportionate emissions generated from the raw material acquisition and manufacturing stages (scope 1 and 2 carbon emissions) of pharmaceutical production as opposed to its use, waste management and end‐of‐life (scope 3 carbon emissions) [51]. For example, quantifying water use (water footprint) across the life cycle of interventions, quantifying waste volumes generated along the care pathway, and human health and biodiversity impacts attributed to the life cycle of interventions [52].

Additionally, due to the nature of EHR data extraction techniques used within this study, we were not able to separate referrals and medication prescriptions that may have resulted from a follow‐up MSK primary care consultation that was not coded for an MSK pain site for example, if an individual was referred to oncology following a non‐MSK coded GP appointment this referral would have appeared in our count data. Furthermore, we did not include individuals who consulted for inflammatory conditions (such as Rheumatoid Arthritis), meaning our results cannot be generalised to all MSK conditions. Finally, we only had access to individual's primary care EHR and their linked survey data, and therefore, we were unable to tell if an individual attended referred outpatient appointments and the frequency of attendance. Similarly, if their care was escalated following an outpatient appointment, they attended accident and emergency, underwent surgical interventions, or were admitted to hospital as an inpatient for their MSK condition, we were unable to capture this data. This limitation means that the true cost and carbon output of MSK condition pathways is likely to be higher than our estimates because these secondary care resources have large economic and environmental outputs.

To enable a more extensive economic and environmental analysis of MSK care pathways, future work should focus on linking individuals' primary and secondary care records to further populate our cost‐carbon model. Additionally, analyses could encompass a broader range of environmental outcomes and clinical resources, such as waste product carbon emissions and include an extended list of medication prescriptions. Furthermore, benchmarking the cost and carbon values for MSK condition management decisions would allow for the standardised comparison between MSK care services. Data linkage projects, such as the MIDAS GP study, could also assess the correlation between participant outcomes and experiences, and cost and carbon output. Trials could also use the methodology developed in this study to investigate how clinician decision support tools and behavioural change strategies impact resource use and their associated economic and environmental output. Finally, we recognise the need for ‘cradle to grave’ and ‘bottom‐up’ process‐based (product) environmental life cycle assessments that evaluate the environmental impact of resources within the carbon model (particularly for pharmaceuticals). This would build on the largely top‐down approach that is currently used and strengthen the accuracy of our carbon output estimates.

We have presented foundational research in the field of environmental and economic analyses of the primary care of MSK conditions within the United Kingdom. The value of this work is its impact to guide the future development of sustainable policy and practice for the care of MSK conditions. Furthermore, it can be used as an insight to help service commissioners, managers, and clinicians understand where the variation in care is occurring. In addition, this novel data will help to support the newly evolving landscape in developing environmental sustainability in health technology assessment. Future work should focus on benchmarking the cost and carbon output of MSK condition management decisions and expanding our analysis to secondary care data.

## Conflicts of Interest

The authors declare no conflicts of interest.

## Supporting information

Supporting Information S1

Supporting Information S2

Supporting Information S3

## Data Availability

The de‐identified, aggregated, practice‐level analysis dataset and statistical code are publicly available on Open Science Framework at https://osf.io/e542w/(doi:10.17605/OSF.IO/E542W).

## References

[1] World Health Organisation “Climate Change,” January 04, 2024, https://www.who.int/news‐room/fact‐sheets/detail/climate‐change‐and‐health#:~:text=Research%20shows%20that%203.6%20billion,diarrhoea%20and%20heat%20stress%20alone.

[2] United Nations, Department of Economic and Social Affairs – Sustainable Development . “The 17 Goals,” July 08, 2024, https://sdgs.un.org/goals.

[3] G. H. Brundtland Report of the World Commission on Environment and Development: Our Common Future (1987) Accessed, 06 20, 2024, https://sustainabledevelopment.un.org/content/documents/5987our‐common‐future.pdf.

[4] World Economic Forum . “Quantifying the Impact of Climate Change on Human Health,” March 11, 2024, https://www3.weforum.org/docs/WEF_Quantifying_the_Impact_of_Climate_Change_on_Human_Health_2024.pdf.

[5] World Health Organisation . “Global Spending on Health: Rising to the Pandemic’s Challenges,” April 02, 2024, https://iris.who.int/bitstream/handle/10665/365133/9789240064911‐eng.pdf?sequence=1.

[6] Met Office . “What Is Climate Change?,” June 19, 2024, https://www.metoffice.gov.uk/weather/climate‐change/what‐is‐climate‐change#:~:text=In%2050%20years'%20time%2C%20by,and%20up%20to%2060%25%20drier.

[7] M. Jakovljevic , M. Jakab , U. Gerdtham , et al., “Comparative Financing Analysis and Political Economy of Noncommunicable Diseases,” Journal of Medical Economics 22, no. 8 (2019): 722–727, 10.1080/13696998.2019.1600523. 30913928

[8] G. Bianco , R. M. Espinoza‐Chavez , pg Ashigbie , et al., “Projected Impact of Climate Change on Human Health in Low‐And Middle‐Income Countries: A Systematic Review,” BMJ Global Health 8, no. 3 (2024): e015550, 10.1136/bmjgh-2024-015550. PMC11733072 39357915

[9] United Nations Climate Change November 2022. “Sharm El‐Sheikh Climate Change Conference,” January 04, 2023, https://unfccc.int/cop27.

[10] L. Rodríguez‐Jiménez , M. Romero‐Martín , T. Spruell , Z. Steley , and J. Gómez‐Salgado , “The Carbon Footprint of Healthcare Settings: A Systematic Review,” Journal of Advances Nursing 79, no. 8 (2023): 2830–2844, https://onlinelibrary.wiley.com/doi/10.1111/jan.15671. 10.1111/jan.15671 37198974

[11] Eurostat – Statistics Explained . Glossary: Global‐Warming Potential (GWP), November 06, 2024, https://ec.europa.eu/eurostat/statistics‐explained/index.php?title=Glossary:Global‐warming_potential_(GWP).

[12] N. Watts , M. Ammann , N. Arnell , et al., “The 2020 Report of the Lancet Countdown on Health and Climate Change: Responding to Converging Crises,” Lancet 397, no. 10269 (2021): 129–170, 10.1016/S0140-6736(20)32290-X. 33278353 PMC7616803

[13] I. Tennison , S. Roschnik , B. Ashby , et al., “Health Care’s Response to Climate Change: A Carbon Footprint Assessment of the NHS in England,” Lancet Planetary Health 5, no. 2 (2021): E84–E92, 10.1016/S2542-5196(20)30271-0. 33581070 PMC7887664

[14] Health Care Without Harm . “Health Care’s Climate Footprint: How the Health Sector Contributes to the Climate Crisis and Opportunities for Action,” April 02, 2024, https://noharm‐global.org/sites/default/files/documents‐files/5961/HealthCaresClimateFootprint_092319.pdf.

[15] H. Ritchie and M. Roser . “C02 Emissions – OurWorldInData.Org,” April 02, 2024, https://ourworldindata.org/co2‐emissions#article‐citation.

[16] The National Health Service England . “Delivering a ‘Net Zero’ National Health Service,” January 04, 2023, https://www.england.nhs.uk/greenernhs/wp‐content/uploads/sites/51/2022/07/B1728‐delivering‐a‐net‐zero‐nhs‐july‐2022.pdf.

[17] World Business Council for Sustainable Development . “The Greenhouse Gas Protocol: A Corporate Accounting and Reporting Standard,” January 04, 2023, https://ghgprotocol.org/sites/default/files/standards/ghg‐protocol‐revised.pdf.

[18] L. Gough , M. Quinn , and D. Donaghy . “Net Zero for Health and Social Care: How Can Regulation Play its Part?,” January 04, 2023, https://blogs.deloitte.co.uk/health/2022/09/net‐zero‐for‐health‐and‐social‐care‐how‐can‐regulation‐play‐its‐part.html#:~:text=Currently%2C%20the%20health%20and%20care%20system%20is%20responsiblereduce%20emissions%20by%20the%20planned%20net%20zero%20date.

[19] “The Kings Fund Activity in This NHS,” April 01, 2023, https://www.kingsfund.org.uk/projects/nhs‐in‐a‐nutshell/NHS‐activity.

[20] J. Nicolet , Y. Meuller , P. Paruta , J. Boucher , and N. Senn , “What Is the Carbon Footprint of Primary Care Practises? A Retrospective Life‐Cycle Analysis in Switzerland,” Environmental Health 21 (2022): 3, 10.1186/s12940-021-00814-y. 34980135 PMC8723904

[21] Versus Arthritis . “The State of Musculoskeletal Health 2023,” January 04, 2023, https://versusarthritis.org/media/duybjusg/versus‐arthritis‐state‐msk‐musculoskeletal‐health‐2023pdf.pdf.

[22] K. P. Jordan , U. T. Kadam , R. Hayward , M. Porcheret , C. Young , and P. Croft , “Annual Consultation Prevalence of Regional Musculoskeletal Problems in Primary Care: An Observational Study,” BMC Musculoskeletal Disorders 11, no. 1 (2010): 144, 10.1186/1471-2474-11-144. 20598124 PMC2903510

[23] I. M. Sajad , A. Parkunan , and K. Frost , “Unintended Consequences: Quantifying the Benefits, Iatrogenic Harms and Downstream Cascade Costs of Musculoskeletal MRI in UK Primary Care,” BMJ Open Qual 10, no. 3 (2021): e001287, 10.1136/bmjoq-2020-001287. PMC8256731 34215659

[24] A. Cieza , K. Causey , K. Kamenov , S. W. Hanson , S. Chatterji , and T. Vos , “Global Estimates of the Need for Rehabilitation Based on the Global Burden of Disease Study 2019: A Systematic Analysis for the Global Burden of Disease Study 2019,” Lancet 396, no. 10267 (2020): 2006–2017, 10.1016/S0140-6736(20)32340-0. 33275908 PMC7811204

[25] R. J. Black , M. Cross , L. M. Haile , et al., “Global, Regional, and National Burden of Rheumatoid Arthritis, 1990‐2020, and Projections to 2050: A Systematic Analysis of the Global Burden of Disease Study 2021,” Lancet Rheumatology 5, no. 10 (2023): e594–e610, 10.1016/s2665-9913(23)00211-4. 37795020 PMC10546867

[26] B. J. McKenzie , R. Haas , G. E. Ferreira , G. Maher , and R. Buchbinder , “The Environmental Impact of Health Care for Musculoskeletal Conditions: A Scoping Review,” PLoS One 17, no. 1 (2022): e0276685, 10.1371/journal.pone.0276685. 36441677 PMC9704655

[27] R. Burgess , J. Hall , A. Bishop , M. Lewis , and J. Hill , “Costing Methodology and Key Drivers of Health Care Costs Within Economic Analyses in Musculoskeletal Community and Primary Care Services: A Systematic Review of the Literature,” Journal of Primary Care & Community Health 11 (2020): 2150131719899763, 10.1177/2150132719899763. PMC6966248 31941391

[28] G. Peat , J. Bailey , K. Jordan , et al., “Multi‐level Integrated Data for Musculoskeletal Health Intelligence and ActionS,” 2024 MIDAS). Accessed:, 04 02, 2024, https://osf.io/vj32d.

[29] H. Birkinshaw and J. C. Hill . “Musculoskeletal Pathways Study ISRCTN Registration,” April 04, 2024, https://www.google.com/search?q=Orthopathways+trial+keele&oq=Orthopathways+trial+keele&gs_lcrpp=EgZjaHJvbWUyBggAEEUYOTIJCAEQIRgKGKABMgklAhAhGAoYoAEyCQgDECEYChigATIJCAQQIRgKGKAB0gEJMTQyMjlqMWo3qAIAsAIA&sourceid=chrome&ie=UTF‐8.

[30] Department for Environmental, Food & Rural Affairs . “Department for Environmental, Food and Rural Affairs Outcome Delivery Plan: 2021 to 2022.” Available: Department for Environment, Food and Rural Affairs Outcome Delivery Plan: 2021 to 2022 ‐ GOV.UK [Accessed: 27/11/2024].

[31] World Resources Institute and World Business Council for Sustainable Development . “Greenhouse Gas Protocol Product Life Cycle Accounting and Reporting Standard,” 2011. Greenhouse Gas Protocol Product Life Cycle Accounting and Reporting Standard | World Resources Institute, https://ghgprotocol.org/sites/default/files/standards/Product‐Life‐Cycle‐Accounting‐Reporting‐Standard_041613.pdf.

[32] Executive Agency for Health and Consumers 2013. “Study on the Environmental Risks of Medical Products,” June 21, 2024, https://health.ec.europa.eu/system/files/2016‐11/study_environment_0.pdf.

[33] M. Berners‐Lee , D. C. Howard , J. Moss , K. Kaivanto , and W. A. Scott , “Greenhouse Gas Footprinting for Small Businesses‐The Use of Input‐Output Data,” Science of the Total Environment 409, no. 5 (2011): 883–891, 10.1016/j.scitotenv.2010.11.023. 21183205

[34] NICE 2020. “Low Back Pain and Sciatica in over 16s: Assessment and Management,” June 20, 2024, https://www.nice.org.uk/guidance/ng59/chapter/Recommendations. 33090750

[35] M. D. Sullivan and C. Q. Howe , “Opioid Therapy for Chronic Pain in the United States: Promises and Perils,” Pain 154, no. Supplement 1 (2013): S94–S100, 10.1016/j.pain.2013.09.009. 24036286 PMC4204477

[36] NICE , Osteoarthritis: Care and Management. Available: Osteoarthritis: Care and Management | Guidance (NICE, 2022), https://www.nice.org.uk/guidance/ng226.

[37] I. Lin , L. Wiles , R. Waller , et al., “What Does Best Practice Care for Musculoskeletal Pain Look like? Eleven Consistent Recommendations From High‐Quality Clinical Practice Guidelines: Systematic Review,” British Journal of Sports Medicine 54, no. 2 (2020): 79–86, 10.1136/bjsports-2018-099878. 30826805

[38] A. Baldini , M. Von Korff , and E. H. B. Lin , “A Review of Potential Long‐Term Opioid Therapy: A Practitioners Guide,” Primary Care Comparison CNS Disorders 14, no. 3 (2012): 14, 10.4088/PCC.11m01326. PMC3466038 23106029

[39] C. Rizan and F. B. Mahmod , “Environmental Impact and Life Cycle Financial Cost of Hybrid (Reusable/single‐use) Instruments versus Single‐Use Equivalents in Laparoscopic Cholecystectomy,” Surgical Endoscopy 36, no. 6 (2021): 4067–4078, 10.1007/s00464-021-08728-z. 34559257 PMC9085686

[40] National Institute for Health and Care Excellence . “Medicines Optimisation: The Safe and Effective Use of Medicines to Enable the Best Possible Outcome,” July 08, 2024, https://www.nice.org.uk/guidance/ng5. 26180890

[41] A. Sharma , A. Singh , M. A. Dar , et al., “Menace of Antimicrobial Resistance in LMICs: Current Surveillance Practises and Control Measure to Tackle Hostility,” Journal of Infection and Public Health 15, no. 2 (2022): 172–181, 10.1016/j.jiph.2021.12.008. 34972026

[42] Department of Health & Social Care , “Good for You, Good for Us, Good for Everybody,” July 08, 2024, https://assets.publishing.service.gov.uk/government/uploads/system/uploads/attachment_data/file/1019475/good‐for‐you‐good‐for‐us‐good‐for‐everybody.pdf.

[43] The Kings Fund 2011. “Variations in Health Care,” July 08, 2024, https://assets.kingsfund.org.uk/f/256914/x/2ceabe804f/variations_health_care_good_bad_inexplicable_2011.pdf.

[44] K. H. Hanson , N. Brikci , D. Erlangga , et al., “The Lancet Global Health Commissions on Financing Primary Health Care: Putting People at the Centre,” Lancet Global Health 10, no. 5 (2022): e715–e772, 10.1016/s2214-109x(22)00005-5. 35390342 PMC9005653

[45] “Google. Environmental Report” 2024, August 10, 2024, https://www.gstatic.com/gumdrop/sustainability/google‐2024‐environmental‐report.pdf.

[46] L. Daniel‐Watanabe , R. Moore , B. Tongue , and S. Royston . “What Is the Carbon Footprint of Digital Healthcare 2024,” June 20, 2024, https://ukerc.rl.ac.uk/UCAT/PUBLICATIONS/UKERC_EnergySHINES_What‐is‐the‐Carbon‐Footprint‐of‐Digital‐Healthcare.pdf.

[47] A. Braybrooke , K. Baraks , R. Burgess , A. Banerjee , and J. C. Hill , “Quality Indicators for the Primary and Community Care of Musculoskeletal Conditions: A Systematic Review,” Archives of Physical Medicine and Rehabilitation (2024), 10.1016/j.apmr.2024.08.022. 39369932

[48] C. Batcup , M. Breth‐Petersen , T. Dakin , et al., “Behavioural Change Interventions Encouraging Clinicians to Reduce Carbon Emissions in Clinical Activity: A Systematic Review,” BMC Health Services Research 23, no. 1 (2023): 384, 10.1186/s12913-023-09370-2. 37081553 PMC10116654

[49] NHS England . “Cancer Services Profiles, 2023 Annual Update,” February 06, 2024, https://digital.nhs.uk/data‐and‐information/publications/statistical/cancer‐services‐profiles/2023‐annual‐update#:~:text=there%20were%204%2C619%20urgent%20suspected,detection%20rate)%20was%2055.6%25.

[50] M. Lynch , G. Peat , K. Jordan , D. Yu , and R. Wilkie , “Where Does it Hurt? Small Area Estimates and Inequality in the Prevalence of Chronic Pain,” European Journal of Chronic Pain 27, no. 10 (2023): 1177–1186, 10.1002/ejp.2148. PMC10947147 37345222

[51] A. G. Parvatker , H. Tunceroglu , J. D. Sherman , et al., “Cradle‐to‐gate Greenhouse Gas Emissions for Twenty Anaesthetic Active Pharmaceutical Ingredients Based on Process Scale‐Up and Process Design Calculations,” ACS Sustainable Chemistry & Engineering 7, no. 7 (2019): 6580–6591, 10.1021/acssuschemeng.8b05473.

[52] C. Rizan , T. Brophy , R. Lillywhite , M. Reed , and M. F. Butta , “Life Cycle Assessment and Life Cycle Cost of Repairing Surgical Scissors,” International Journal of Life Cycle Assessment 27, no. 6 (2022): 780–795, 10.1007/s11367-022-02064-7.

