# Supplementary material for: Estimating the Cost and Carbon Output of Musculoskeletal Primary Care Management Decisions: A Retrospective Analysis of Electronic Health Records
Source: Int J Health Plann Manage. 2025 Mar 22;40(4):907–22. doi: 10.1002/hpm.3919 (PMC12215594; doi:10.1002/hpm.3919)
Supplement: Supplementary file 2 — Supporting Information S2 [file HPM-40-907-s001.docx]

# **Supplementary file 2 – Targeted literature search**

# **Methods**

Multiple non-systematic targeted literature searches were carried out to identify the current evidence surrounding MSK pathways. These searches were split across 5 areas. The aim of the 5 searches were to:

- Understand the resource use within current MSK pathways.
- Extract information on previous economic evaluations in MSK pathways and the type of modelling which was used.
- Highlight examples of previous environmental evaluations in MSK, including the type of modelling and resources captured in these evaluations.
- Extract potential inputs to be used in the model for the environmental impact aspect.
- Extract potential inputs to be used in the model for the economic cost aspect.

The searches were not intended to be exhaustive or highly sensitive. They were instead designed to retrieve a representative range of relevant studies, guidelines, and model inputs.

The selected pathways to be captured within the model were decided between Primum Digital Ltd and key clinical advisors. This was based on the number of people expected to be captured in the pathway, as well as the impact that CrossCover OrthoPathway is likely to have.

## **Eligibility Criteria**

To identify relevant evidence to answer the review question, a clear definition of eligible studies was developed. This eligibility criteria outlined below applies to all searches. The eligibility criteria were not applied to the targeted searches for environmental and economic inputs, given that the targeted searches focused on standard health economic literature, while a wider range was sought to capture environmental specific inputs.

### **Population**

The criteria applied to identify eligible studies included the following:

- Adults (16 and over), with no restrictions on gender or race.
- MSK pathways of interest:
  - Neck
  - Shoulder
  - Back
  - Hip
  - Elbow
  - Wrist or hand
  - Knee
  - Foot or ankle

### **Interventions**

There were no restrictions in relation to interventions.

### **Comparators**

There were no restrictions in relation to comparators.

### **Outcomes**

The key outcomes to be captured within the review are:

- Evidence of the resource use associated with MSK pathways.
- Previous economic and environmental evaluations of MSK pathways. This is to be used to inform the current model development of CrossCover OrthoPathway.
- Cost and environmental inputs which are to be used for the modelling of the MSK pathways.

### **Study Design**

Study designs included those containing economic, environmental, or costing evaluations for the disease areas of interest (MSK pathways). This included those undertaking or reporting cost of illness, burden of illness, economic modelling or costing, or costs relating to health and social care systems.

To understand the current care pathway, published clinical guidelines were used, rather than specific studies.

### **Limits**

Studies and guidelines were selected for the time span from 2010 onwards and published as full text articles in English. Only studies from populations in the United Kingdom (UK) were considered, other than for the environmental evaluations, where the searches were not limited to the UK.

Studies published in abstract form only, conference presentations, editorials, news items and short correspondence were not considered within the results.

## **Pathway Resource Use**

To evaluate the current MSK pathways, we conducted pragmatic, targeted literature searches. The searches were conducted using a range of resources, including:

- NICE guidance webpages (<https://www.nice.org.uk/guidance>)
- PubMed (<https://www.pubmed.ncbi.nlm.nih.gov/>)
- Google Scholar (<https://www.scholar.google.com/>)
- Google (<https://www.google.com/>)

These resources included sources of journal literature and grey literature.

The searches of the NICE guidance webpages retrieved a number of potentially relevant documents. These include clinical guidance and economic reviews (including economic evidence previously submitted to NICE). Details of the key resource uses were extracted for each of the pathways. The search of PubMed used strategic Boolean operators to target the results. The PubMed search strategy is detailed in Appendix A, Figure A 1.

## **Previous Economic Evaluations: MSK**

To evaluate previous economic evaluations of interventions within MSK, a pragmatic targeted literature search was carried out. Searches for economic evaluations were limited to orthopaedic wide interventions. This is because specific conditions within orthopaedics would not be similar enough to the evaluation of CrossCover OrthoPathway, which has a much wider population. Hence, searches were focused on interventions which are likely to be system wide or pathway changing to orthopaedics in general, to inform the model development.

The search was made on PubMed and the search strategy is detailed in Appendix A, Figure A 2. Further searches were conducted through Google Scholar, Google, and grey literature such as economic evaluations captured as part of systematic literature reviews (SLRs).

## **Previous Environmental Evaluations: MSK**

To evaluate the current evidence surrounding the environmental impact of MSK and orthopaedic health care, a further pragmatic targeted literature search was conducted. Searches were not limited to the UK for this search, as YHEC expected less evidence to be generated compared to standard economic evaluations, so a wider approach to searching was taken. The search was carried out on PubMed and the search strategy is detailed in Appendix A, Figure A 3. Further searches were also conducted through Google Scholar, Google, and grey literature (such as sources of the returned studies).

## **Environmental Inputs**

A targeted literature search was conducted to identify the environmental impact of the expected resource use associated with MSK pathways. This targeted search was conducted separately, focusing on environmental inputs, rather than specific environmental analysis of the MSK pathway. This was largely conducted through more rapid searches on Google and Google Scholar to inform input values, such as average carbon emissions from GP appointments and outpatient appointments. Previous evaluations for MSK pathways were also used to extract potential environmental inputs.

To determine more specific environmental inputs such as those from diagnostic tools, a further targeted search was carried out on PubMed using the terms “carbon emissions” and “diagnostic imaging”. This search strategy is detailed in Appendix A, Figure A 4.

## **Economic Inputs**

A targeted literature search was conducted to identify costs that would be relevant to the economic modelling. This would be to capture specific inputs that are not contained within other sources, such as any previous MSK economic evaluations. The sources searched as part of this targeted searching were standard published sources used in the economic evaluation of healthcare. These sources are consistent with the NICE methods guide [1] on using costs and resources in economic evaluation. This includes, but is not limited to:

- NHS Cost Collection Data [2]
- The Personal Social Services Research Unit (PSSRU) unit costs [3]
- The electronic market information (eMIT) [4]
- The British National Formulary (BNF) [5]

**References**

1. National Institute for Health and Care Excellence. NICE health technology evaluations: the manual [PMG36]. 2022. Available from: <https://www.nice.org.uk/process/pmg36/chapter/introduction-to-health-technology-evaluation>.

2. NHS England. National Schedule of NHS Costs 2020/21. 2022. Available from: <https://www.england.nhs.uk/publication/2020-21-national-cost-collection-data-publication/>.

3. Personal Social Services Research Unit (PSSRU). Unit Costs of Health and Social Care 2021/2022. 2022. Available from: <https://www.pssru.ac.uk/pub/uc/uc2022/Unit_Costs_of_Health_and_Social_Care_2022.pdf>.

4. Department of Health and Social Care Drugs and pharmaceutical electronic market information tool (eMIT): national database. 2022. Available from: <https://www.gov.uk/government/publications/drugs-and-pharmaceutical-electronic-market-information-emit>

5. National Institute for Health and Care Excellence. Briitsh National Formulary. Available from: <https://bnf.nice.org.uk/>.

Appendix A

Figure A 1 Search strategy: PubMed – pathways search

Search date: 13 February 2023

Retrieved records: 95

Search

("orthopaedic"[All Fields] OR "orthopedics"[MeSH Terms] OR "orthopedics"[All Fields] OR "orthopedic"[All Fields] OR "orthopaedical"[All Fields] OR "orthopedical"[All Fields] OR "orthopaedics"[All Fields]) AND ("critical pathways"[MeSH Terms] OR ("critical"[All Fields] AND "pathways"[All Fields]) OR "critical pathways"[All Fields] OR ("care"[All Fields] AND "pathway"[All Fields]) OR "care pathway"[All Fields]) AND "UK"[All Fields] AND ("adult"[MeSH Terms] OR "adult"[All Fields] OR "adults"[All Fields] OR "adult s"[All Fields])

Translations

Orthopaedic: "orthopaedic"[All Fields] OR "orthopedics"[MeSH Terms] OR "orthopedics"[All Fields] OR "orthopedic"[All Fields] OR "orthopaedical"[All Fields] OR "orthopedical"[All Fields] OR "orthopaedics"[All Fields]

care pathway: "critical pathways"[MeSH Terms] OR ("critical"[All Fields] AND "pathways"[All Fields]) OR "critical pathways"[All Fields] OR ("care"[All Fields] AND "pathway"[All Fields]) OR "care pathway"[All Fields]

adults: "adult"[MeSH Terms] OR "adult"[All Fields] OR "adults"[All Fields] OR "adult's"[All Fields]63 55 or 62 (1551)

Figure A 2 Search strategy: PubMed – economic evaluations

Search date: 17 February 2023

Retrieved records: 80

Search

("cost effectiveness analysis"[MeSH Terms] OR ("cost effectiveness"[All Fields] AND "analysis"[All Fields]) OR "cost effectiveness analysis"[All Fields] OR ("cost"[All Fields] AND "effectiveness"[All Fields]) OR "cost effectiveness"[All Fields]) AND ("orthopaedic"[All Fields] OR "orthopedics"[MeSH Terms] OR "orthopedics"[All Fields] OR "orthopedic"[All Fields] OR "orthopaedical"[All Fields] OR "orthopedical"[All Fields] OR "orthopaedics"[All Fields]) AND ("pathway"[All Fields] OR "pathway s"[All Fields] OR "pathways"[All Fields])

Translations

cost effectiveness: "cost-effectiveness analysis"[MeSH Terms] OR ("cost-effectiveness"[All Fields] AND "analysis"[All Fields]) OR "cost-effectiveness analysis"[All Fields] OR ("cost"[All Fields] AND "effectiveness"[All Fields]) OR "cost effectiveness"[All Fields]

orthopaedic: "orthopaedic"[All Fields] OR "orthopedics"[MeSH Terms] OR "orthopedics"[All Fields] OR "orthopedic"[All Fields] OR "orthopaedical"[All Fields] OR "orthopedical"[All Fields] OR "orthopaedics"[All Fields]

pathway: "pathway"[All Fields] OR "pathway's"[All Fields] OR "pathways"[All Fields]

Figure A 3 Search strategy: PubMed – environmental evaluations

Search date: 17 February 2023

Retrieved records: 23

Search

("orthopaedic"[All Fields] OR "orthopedics"[MeSH Terms] OR "orthopedics"[All Fields] OR "orthopedic"[All Fields] OR "orthopaedical"[All Fields] OR "orthopedical"[All Fields] OR "orthopaedics"[All Fields]) AND (“environmental impact”)

Translations

Orthopaedic: "orthopaedic"[All Fields] OR "orthopedics"[MeSH Terms] OR "orthopedics"[All Fields] OR "orthopedic"[All Fields] OR "orthopaedical"[All Fields] OR "orthopedical"[All Fields] OR "orthopaedics"[All Fields]

Figure A 4 Search strategy: PubMed – environmental inputs

Search date: 20 February 2023

Retrieved records: 2

Search

("carbon emissions”) AND (“diagnostic imaging”)
