# Supplementary material for: Estimating the Cost and Carbon Output of Musculoskeletal Primary Care Management Decisions: A Retrospective Analysis of Electronic Health Records
Source: Int J Health Plann Manage. 2025 Mar 22;40(4):907–22. doi: 10.1002/hpm.3919 (PMC12215594; doi:10.1002/hpm.3919)
Supplement: Supplementary file 3 — Supporting Information S3 [file HPM-40-907-s003.docx]

| Self-Management resource calculations from the survey | |
| --- | --- |
| Resource | Assumption / Calculation |
| Online information / advice carbon emissions | - If used at 3 months but not 6 months count value = 1. - If used at 3- and 6-months count value = 2 - Assumed the patient is referring to different online info |
| Information leaflet carbon emissions | - If used at 3 months but not 6 months count value = 1. - If used at 3- and 6-months count value = 2 - Assumed the patient is referring to a different info leaflet |
| Peer support group carbon emissions | - If used at 3 months but not 6 months count value = 1. - If used at 3- and 6-months count value = 2 - Assumed the patient is still going to the support group – although emissions are per session, but we don’t have this data |
| Exercise: outdoor / home exercise carbon emissions | - If used at 3 months count value = 1. - If used at 6 months count value = 2. - kgC02e = 0.00 |
| Equipment, aids or adaptations carbon emissions | - If used at 3 months but not 6 months count value = 1. - If used at 3- and 6-months count value = 1 - Assumed they are using the same aid |
| Brace, support or splint carbon emissions | - If used at 3 months but not 6 months count value = 1. - If used at 3- and 6-months count value = 1 - Assumed they are using the same brace / splint |
| Vitamins or supplements carbon emissions | - If used at 3 month but not 6 months count value = 1. - If used at 3- and 6-months count value = 2 - Unfortunately, don’t have access to dosage or frequency data, as collected through patient survey |
